# Electrolyte-driven cathode catalysis chemistry in sodium–chlorine batteries

**DOI:** 10.1093/nsr/nwaf579

**Published:** 2025-12-18

**Authors:** Lei Ye, Xiaodong Chen

**Affiliations:** Innovative Centre for Flexible Devices (iFLEX), Max Planck−NTU Joint Laboratory for Artificial Senses, School of Materials Science and Engineering, Nanyang Technological University, Singapore; Innovative Centre for Flexible Devices (iFLEX), Max Planck−NTU Joint Laboratory for Artificial Senses, School of Materials Science and Engineering, Nanyang Technological University, Singapore

The pursuit of sustainable and high-performance energy storage systems has positioned rechargeable sodium-chlorine (Na−Cl_2_) batteries as a promising candidate, leveraging the high theoretical capacity (1200 mAh/g) and natural abundance of core components [1,2]. However, their practical application has long been impeded by poor reversibility and sluggish reaction kinetics, caused by complex side reactions with the electrolyte [3]. To address these issues, fluorine (F)-containing additives such as NaFSI and NaTFSI have been widely adopted, under the assumption that they stabilize the sodium (Na) metal anode by forming a fluorinated solid-electrolyte interphase (SEI), similar to the mechanism established in lithium-based systems [4–6]. However, the strong Lewis acidity of AlCl_3_ in the chloroaluminate electrolyte challenges this paradigm, as it can alter the chemistry of fluorinated additives.

**Figure 1.**
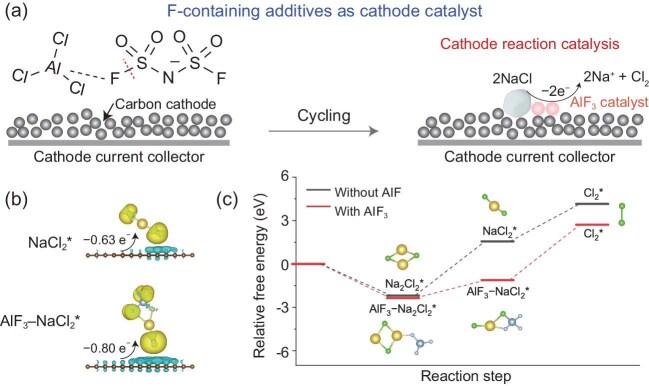
Schematic illustration of the working mechanism of FSI^−^ in a Na–Cl_2_ battery. (a) FSI^−^ reacts spontaneously with AlCl_3_ in the chloroaluminate electrolyte to form AlF_3_ on the carbon cathode, which catalyzes NaCl oxidation. (b) Differential charge density distributions of the adsorbed reaction intermediates on the graphene without (top) and with AlF_3_ (bottom). (c) Calculated Gibbs free energies of the NaCl oxidation reaction on the graphene with and without AlF_3_. Reproduced with permission from ref. [7].

In a recent work, Sun *et al*. revisit the functioning mechanism and reveal a previously overlooked spontaneous chemical transformation that redefines the role of fluorinated additives from anode stabilizers to precursors of active cathode catalysts (Fig. 1a) [7]. They first systematically investigate the effect of F-containing electrolyte additives on Na metal anodes. Contrary to conventional expectations, NaCl rather than NaF is identified as the dominant SEI component. Meanwhile, the additives exhibit negligible influence on Na plating/stripping reversibility. These interesting results motivate the authors to further explore the true role of F-containing electrolyte additives. Using nuclear magnetic resonance (NMR) and high-resolution mass spectrometry (HRMS), the authors identify a complete cleavage of the S–F bond in FSI⁻ or TFSI⁻, forming AlCl_3_F⁻ intermediates through a sulfur(VI) fluoride exchange (SuFEx)-type mechanism. These intermediates further react to produce AlF_3_, which migrates and deposits on the carbon cathode (Fig. 1a). The *in situ* formed AlF_3_ functions as a Lewis-acidic catalyst at the cathode side. Specifically, differential charge density mapping and Bader charge analyses reveal that the formation of the AlF_3_–NaCl_2_* complex facilitates electron transfer between the carbon cathode and NaCl, thereby promoting the cleavage of the Na–Cl bond (Fig. 1b). Moreover, the presence of AlF_3_ lowers the Na_2_Cl_2_* oxidation barrier by 66%, which facilitates the thermodynamically favorable oxidation of NaCl to Cl_2_ (Fig. 1c). These findings indicate that fluorine-containing additives can generate AlF_3_ species on the cathode surface, which in turn accelerates the oxidation kinetics of NaCl and enhances Cl_2_ evolution.

The significance of this mechanistic revelation is twofold. First, it clarifies the electrolyte chemistry of Na–Cl_2_ batteries and provides new insight into the actual role of fluorinated additives. Second, it provides a new materials design principle for high-performance energy storage systems. Guided by this mechanistic insight, the authors develop two cathode engineering strategies: direct incorporation of AlF_3_ into the carbon host and an optimized approach using a polymerized ionic liquid containing FSI⁻ to regulate the generation and spatial distribution of AlF_3_. Beyond the Na–Cl_2_ system, this study introduces a universal ‘additive–cathode catalyst’ strategy, demonstrating how conventional electrolyte additives can be chemically reprogrammed into active catalytic species through rational design. This concept opens a new avenue for developing high-rate and long-life energy storage solutions, including other metal-chlorine and potentially metal-air or metal-sulfur batteries.
